# Endarteritis Deformans

**Published:** 1897-08

**Authors:** E. B. Jackson

**Affiliations:** Houston, Texas


### TEXAS MEDICAL JOURNAL.

ESTABLISHED JULY, 1885.

PUBLISHED MONTHLY.—SUBSCRIPTION $2.00 A YEAR.

VOL. XIII. AUSTIN, AUGUST, 1897. NO. 2.

### Original Contributions.

For the Texas Medical Journal.

### ENDARTERITIS DEFORMANS.

BY E. B. JACKSON, M. D. HOUSTON, TEXAS.

THIS important, interesting, and common lesion of the arterial system, has been described under various names, most of which have, however, been applicable more properly to some stage of the disease. The names referred to are, arterial sclerosis, fibrosis, atheroma, and calcareous degeneration.

Two cases, varying widely with reference to age, coming under the writer's observation within the past two years, serve to illustrate two important points concerning the disease. One case, a man forty-nine, whose physical appearance would indicate that he might be twenty years older, has been intemperate in eating and drinking; has been a house painter; has had specific disease, and has been therefore subjected to all the ætiological factors but one, namely, Bright's disease. His radial artery is hard and chord-like, and rolls and thumps under the examining finger; the brachial artery is tortuous, tense, and visibly pounces up and down with each pulsation of the blood-wave; the arteries of the temporal and carotid regions are likewise visibly disturbed in their effort to promote the transmission of blood; the heart is tumultuously engaged in its endeavor to force the blood around the circuit on time; its stroke is tremendous. Such violent energy is required on account of the contracted lumina, and tortuous courses, of the affected vessels.

There is a long systolic murmur, and the heart is plainly, considerably, hypertrophied. The patient will not forego his intemperate habits, remaining usually in a state of semi-intoxication—failing to comply with measures suggested—and his case has, therefore, not yet been improved.

The second case, a man sixty-eight, who has lived a temperate life, gives no specific history; has not the gouty habit; has not albumen in the urine, and is healthful in appearance. His case is probably the result of an inherited tendency, in which atrophic changes herald the, rather early, approach of senile decay. His radial artery rolls under the finger, and some of the smaller vessels fold up, and protrude with each impulse of the heart-beat, not unlike the coiling motion of the caterpillar when actively engaged in locomotion. Here is evidently a sufficient amount of impingement on the caliber of the vessels of both extremities to warrant the belief that imperviousness, with complete obstruction, and consequent gangrene, may at any moment supervene. The extra amount of work imposed upon the heart, in driving the blood through inelastic, contracted, and tortuous vessels, has, as in all cases, resulted in its hypertrophy, loss of firmness and regularity of rhythm. The sphygmographic tracings are slanting, short and blunt; due, of course, to the slow and incomplete expansion of the thickened arteries.

In the incipiency of this disease, it is said that the muscular coat of the vessel first becomes weakened, which condition is soon followed by a thickening of the inner coat, produced by cell proliferation; the morbid process invading eventually all of the three coats. The organization of cell substances in the vessels' wall, tends toward the obliteration of its lumen by the bulging of the tunica intima upon the blood current; thus narrowing the stream, and stricturing the channel.

It appears that arterial sclerosis would aptly name this stage. The above mentioned deposits of cellular elements may now undergo fatty degeneration—become yellow, and pasty, and soft enough along the inner coat of the vessel to be actually washed away by the blood; leaving in such places only the media and adventitia of the artery. It is easy to see, when dislodged excressences begin to flow in the blood, that the condition is, at once, favorable to thrombosis and embolism—particularly, insomuch, as the vessels are already narrowed.

Necrotic particles often exist in the blood of the aged, hence the frequency of senile gangrene. When a vessel has had its inner coat proliferated, degenerated, and then swept away by the blood, it is readily seen that the maimed portion of it may easily dilate, sacculate, or be bulged into an aneurysm, and ultimately, rupture.

There is still another change which this proliferated substance may undergo — that known as calcification — in which small plates, of concrete formation, resembling spiculæ of bone, are manufactured and lodged in the vessels' walls, with now and again, their sharp edges projecting into the blood stream. Calcific deposits may, in some cases, form complete rings around the smaller vessels.

The disease renders the arteries so weak and brittle in texture that they are liable to rupture without having become dilated or aneurysmal. Such accidents usually occur in the brain, where the vessels are thin, or in the coronary arteries supplying the heart, which are subjected to great commotion and strain. Ill-nutrition of the structures along the distribution of a diseased vessel is sure to be superinduced—cerebral softening, nephritic, cardiac and other organic disturbances thus ensuing. The calcareous plates, when projecting into a vessel, may collect enough fibrin from the blood to plug the vessel, which is another means of producing gangrene. There are many accidents and complications which may follow rapidly, one upon the other; still, it is consoling to remark that it is by no means impossible for a patient to live comfortably, and for a long time, without any further disturbance than the mental worry attendant upon the knowledge of being possessed with an ailment which can not be cured.

Many aged persons have been afflicted with the disease for years, never becoming aware of the fact. Autopsies, which have been held looking to the discovery of other and remote pathologies, have revealed the calcific plates, thick in the aorta and elsewhere, when during life there was no evidence of such disease existing.

Alcoholism, Bright's disease, gout, lead poisoning and specific diseases are, as before intimated, the prime factors in the etiology of endarteritis deformans, i. e.: if the individual has been so situated that his blood vessels have sustained much overstrain through periods of severe and oft repeated muscular pressure upon them. Impure and thick blood flows heavily through the capillaries, whereby a constant state of partial stasis obtains, increasing the tension of the arterial system. Bright's disease keeps the renal arteries engaged on account of the impairment of the capillary tufts, and they are thus occasionally rendered in the course of time atheromatous. If the coronary arteries become affected, the heart-muscle immediately begins to suffer degeneration, changes, becoming atrophic, and likewise very much deranged in function. The hepatic artery is almost never affected with the disease.

In the treatment of endarteritis the patient should be enjoined to abstain from all muscular strain, should never indulge in violent exercise, should be removed to a quiet retreat where the surroundings are such as to exempt from any form of emotional excitement, should be prohibited from imbibing large quantities of liquids, whether water, cider, beer, and particularly alcoholic preparations, thus preventing hyper-distension of the general circulatory system.

Excessive indulgence in nitrogenous foods should be religeously interdicted, as tending to befoul the blood with gouty detritus.

Immoderate indulgence in the relation of the sexes should be impressively counselled against.

If the case occurs in the person of a lead-worker, he should be forthwith restricted from such exposure, and put upon constitutional treatment for lead-poisoning. If the disease is met with in the syphilitic, specific remedies are to be immediately prescribed. If the patient be, plainly, of the gouty habit, a line of systematic and frequent purgation should be inaugurated. For this purpose it is probably best to recommend hunyadi janos water, or compound jalap powder, every day until a considerable depletion is witnessed. The diet should consist chiefly of farinaceous food and succulent vegetables. If the heart should grow perceptibly weaker under this regimen, the physician may proceed at once to administer, hypodermically, one hundredth grain doses of nitro-glycerine four or five times a day, or even much oftener when really necessary. This remedy is supposed to have a two-fold effect on the disease under discussion, namely —it promotes renewed energy in the heart-beat, and relieves the tension throughout the arterial tree by relaxing the vessel walls.

Long continued exertions of the brain are highly injurious, and should be so considered in summing up a line of treatment. Insomnia and mental anguish very materially favor the progress of the disease, insomuch as they very quickly superinduce gastric, hepatic and intestinal inactivity, followed in good time by dyscrasia of assimilation, and ultimately by a state of progressive mal-nutrition.

The patient becomes now sad-eyed, sad-complexioned, and is, indeed, the personification of misery.

Prevention of endarteritis deformans should be the supreme aim of every physician seeing evidences of its approach, since its cure, after reaching an advanced stage, can not, without great strain to the conscience, be promised.

